# Preparation of Diopside-Rich Compounds Using Rice Husks and Their Phosphorus Recovery Capability

**DOI:** 10.3390/bioengineering12121339

**Published:** 2025-12-08

**Authors:** Ryotaro Seki, Hiyu Naka, Ryouta Umebayashi, Kay Teraoka, Toru Nonami

**Affiliations:** 1 Faculty of Engineering, Chukyo University, 101-2 Yagoto Honmachi, Showa-ku, Nagoya 466-8666, Japan; t12515m@m.chukyo-u.ac.jp (R.S.); hiyuu123107@gmail.com (H.N.); ryota_0315@icloud.com (R.U.); 2 National Institute of Advanced Industrial Science and Technology, 1-1-1 Higashi, Tsukuba 305-8566, Japan

**Keywords:** rice husk, silica, diopside, phosphorus recovery

## Abstract

Rice husks, wastes from rice production, are expected to be useful as a phosphorus recovery material. While rice husks could be used for phosphorus recovery as they are, there is potential to increase their phosphorus recovery capacity by diopside conversion. This is because diopside is one of the silica-based biomaterials that has the characteristic of precipitating calcium phosphate from aqueous solutions containing phosphorus, such as body fluids. In this study, diopside was synthesized by immersing Aichi no Kaori rice husks in a Ca–Mg aqueous solution followed by calcination. The diopside content of resulting compound was 12%. Phosphorus recovery by the diopside-containing compound was confirmed. Given the diopside content in the compound, it was considered possible that components other than diopside might also be contributing to the phosphorous recovery.

## 1. Introduction

Rice is one of the world’s three major staple grains and serves as a primary food source for a significant portion of the global population. However, approximately 20% of rice production consists of husks, which are often treated as waste [1]. For instance, Japan’s rice production in 2023 was about 7.2 million tons, generating approximately 1.4 million tons of rice husks [2]. Historically, rice husks were disposed of by open burning; however, concerns regarding the environmental impact of the emitted gases have led to the establishment of global regulations restricting this practice.

Given these environmental concerns, there is growing interest in utilizing rice husks as a resource. A key characteristic of rice husks is their high content of fine amorphous silica, which exhibits good reactivity. Rice husk-derived silica is used in industrial applications such as tire fillers [3], cement components [4,5], and semiconductor encapsulants [6]. It is also employed in applications requiring high purity of silica; these include its use as a raw material for SiC whiskers [7] and in the production of high-purity metallurgical grade silicon [8]. Furthermore, rice husk silica serves as a raw material for zeolite synthesis [9]. Although approximately two-thirds of the rice husks are currently being repurposed for such applications [10], their further promotion as a resource would be beneficial.

Rice husks are expected to be useful as a phosphorus recovery material, offering avenues for expanding rice husk applications. Phosphorus is a useful resource, commonly used in fertilizers, surfactants, and livestock feed additives. However, a significant portion of the phosphorus used in these applications is not fully consumed and is instead discharged into the hydrosphere through wastewater. While phosphorus is essential for the growth of bacteria, plants, and animals in aquatic ecosystems, excessive levels can lead to the overgrowth of blue-green algae because they feed on phosphorus, eventually depleting dissolved oxygen and causing the death of fish and other aquatic life. This also affects human populations that rely on lakes and marshes as water sources. Despite its low concentration in natural water bodies compared to phosphate ore, phosphorus dispersed in the hydrosphere is relatively easy to access. Various methods have been proposed to recover it, including the precipitation of phosphorus as calcium phosphate using carbonized rice husk–calcite composites [11].

Diopside has also emerged as a promising silica-based material for phosphorus recovery. As a compound within the CaO-MgO-SiO_2_ system, diopside has been found to promote the precipitation of calcium phosphate, such as apatite, on its surface when in contact with body fluids [12]. The idea is to use this feature to recover phosphorus in aqueous environments. Diopside is typically synthesized through the reaction of silica with calcium and magnesium. Various synthesis methods have been reported, including solid-state reaction, sol–gel method, coprecipitation, and spray pyrolysis deposition techniques [13]. Since 1995, our research group has been synthesizing diopside using the sol–gel method, focusing on its ability to promote bone formation, that is, calcium phosphate precipitation on its surface [14]. More recently, studies have demonstrated that diopside synthesized via the sol–gel method can be utilized for phosphorus recovery from aqueous solutions [15].

Rice husks have shown potential as a silica source for diopside synthesis [16,17,18]. However, reproducing previous research findings requires careful selection of rice husk samples. Since rice husks, like rice grains, exhibit variability based on geographic origin and cultivar, their composition can differ significantly. At the same time, in the context of waste utilization, cost efficiency is a key consideration. Ideally, rice husks should be procured locally for local consumption—sourcing specific rice husks from distant locations may not be practical. Therefore, in this study, we synthesized diopside using rice husks from *Aichi no Kaori*, a widely cultivated rice variety in Aichi Prefecture, where our university is located, and evaluated its phosphorus recovery capability.

## 2. Materials and Methods

### 2.1. Synthesis

Rice husks from the 2018 harvest of Aichi no Kaori were used in this study. *Aichi no Kaori* is a Japonica rice cultivar developed in 1977 by the Aichi Agricultural Research Center through the cross-breeding of *Hatsushimo*, a rare rice variety from Aichi, and *Minenoasahi*, a strain of *Koshihikari*. This cultivar accounts for the largest percentage of rice produced in Aichi Prefecture [19].

Diopside was synthesized using rice husk-derived silica by immersing 3 g of rice husks in 120 mL of an aqueous solution containing equimolar amounts of calcium and magnesium ions (Ca–Mg aqueous solution), followed by drying and calcination. The Ca–Mg aqueous solution was prepared by mixing aqueous solutions of calcium chloride dihydrate and magnesium chloride hexahydrate at concentrations of 0.25 mol/L and 2.0 mol/L in equal proportions. The rice husks were immersed in this solution at 25 °C for 6 h under continuous shaking. The rice husks after the immersion were dried at 80 °C for at least 48 h, followed by calcination in an electric furnace (NL-2025D, Motoyama, Osaka, Japan). Calcination was performed at 600, 800, 1000, and 1300 °C at a heating rate of 18 °C/min and retention time of 2 h. The calcined rice husks were then ground in an agate mortar and passed through a sieve with a 250-micron opening. The name of each sample was “Ca–Mg aqueous solution concentration-calcination temperature”.

The crystalline phase of the synthesized samples was identified using powder XRD (Empyrean, Malvern Panalytical, Malvern, UK). XRD measurements were conducted at a scan angle range of 20.0130–69.9850°, scan step size of 0.0260°/min, scan step time of 27.54 s, tube voltage of 45 kV, and current of 40 mA. Diopside content ratio of the diopside-containing sample was calculated by the Reference Intensity Ratio (RIR) method.

### 2.2. Phosphorus Recovery Test

Among the synthesized samples, phosphorus recovery tests were conducted on those in which diopside formation was confirmed. The phosphorus recovery target solution was prepared using potassium dihydrogen phosphate (reagent special grade, Wako Pure Chemicals, Osaka, Japan) dissolved in ultrapure water at a concentration of 0.01 mol/L.

The phosphorus recovery ability of diopside was evaluated based on the rate of decrease in phosphate ion concentration in the target solution using the molybdenum blue method. The experimental procedure was as follows: 300 mg of rice husk-derived diopside was added to 300 mL of the phosphorus-containing solution. The mixture was stirred for 30 min and then recovered. The treated solution was filtered using a syringe filter with a pore size of 0.20 µm (DISMIC-25H, Toyo Roshi Kaisha, Tokyo, Japan). The filtrate was mixed with an ascorbic acid solution and a molybdic acid solution at a ratio of 25:1 and stirred for 15 min. The absorbance of the treated solution was measured at 883 nm using a spectrophotometer (U-5100, Hitachi High-Tech, Tokyo, Japan). The phosphate ion reduction ratio in the solution was determined from a calibration curve.

The crystalline phase of the sample after the test was identified using powder XRD (MiniFlex, Rigaku, Tokyo, Japan). The XRD measurements were conducted at a scan angle range of 3–90°, scan speed of 3°/min, tube voltage of 30 kV, and current of 15 mA.

### 2.3. Morphological Observation

The morphology of the samples was observed by SEM (S-2600N, Hitachi High-Tech, Tokyo, Japan).

## 3. Results

**Figure 1:**
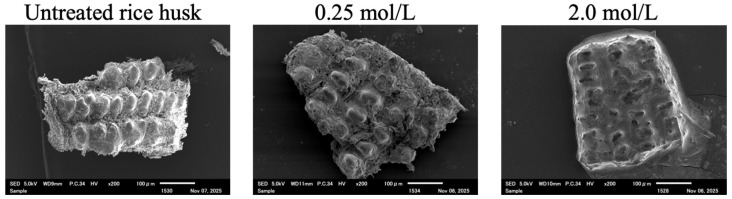
The SEM images of the untreated rice husk, and those immersed in the Ca–Mg aqueous solutions. Numerals at the top of the images indicate the concentrations of the Ca–Mg aqueous solutions.

Figure 1 shows SEM images of the untreated rice husk (left), and those immersed in Ca–Mg aqueous solutions and dried (center and right). Apparently, the covering substance was observed on the rice husk treated with Ca–Mg aqueous solutions at 2.0.

**Figure 2:**
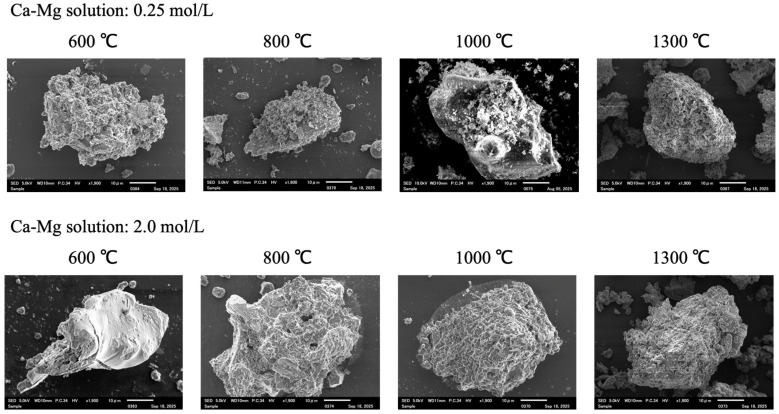
The SEM images of the synthesized samples.

Figure 2 shows SEM images of the calcinated rice husks treated with the Ca–Mg aqueous solutions (synthesized samples). While there are no clear differences in appearance between the SEM images of the samples, it could be said that the contours of the constituent particles of the samples calcined at 1300 °C appear more distinctive. Further, fibrous matter suggesting organic charcoal residues were found in the 2.0-600 sample.

**Figure 3:**
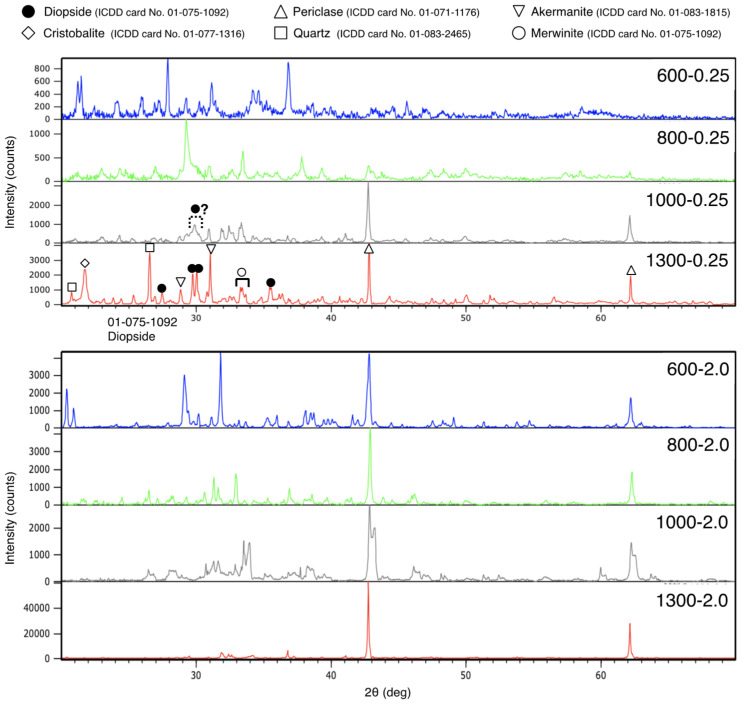
The XRD patterns of the synthesized samples. “⚫︎?” in the diffraction pattern of 1000-0.25 sample indicates peaks that may correspond to diopside.

Figure 3 shows the X-ray diffraction (XRD) patterns of the synthesized samples. The diffraction peak of diopside was confirmed in the diffraction pattern of 0.25-1300. The diffraction pattern of 0.25-1300 also indicated the presence of cristobalite, quartz and periclase. Furthermore, diffraction peaks were observed near 29°, 31° and 33–34°. Although these peaks could not be definitively identified based on the results of this study, the former two peaks were considered to correspond to akermanite, and the latter one was considered to correspond to merwinite. The diopside content of 0.25-1300 was 12%.

Diopside diffraction peaks were not observed in the XRD patterns of samples other than 0.25-1300. In the 0.25-1000 XRD pattern, a broad peak near 30 degrees is possibly attributed to diopside, which is not identifiable from this measurement.

**Figure 4:**
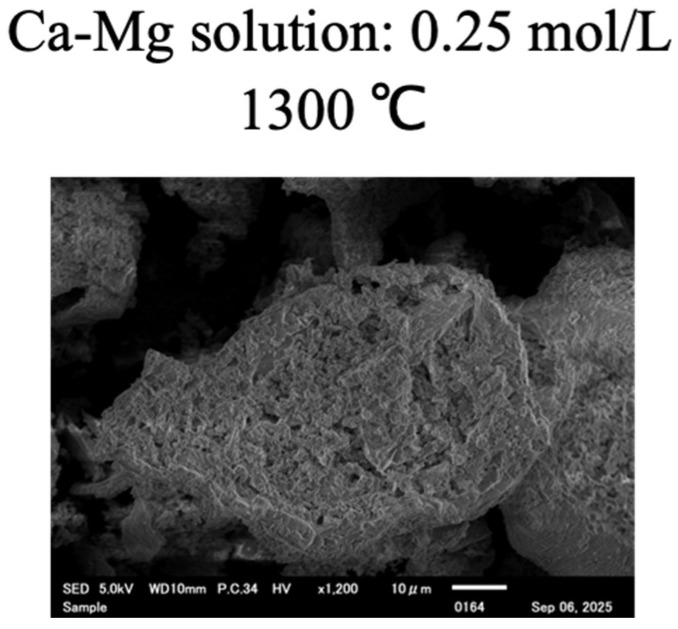
The SEM image of the synthesized samples after the phosphorus recovery test.

**Figure 5:**
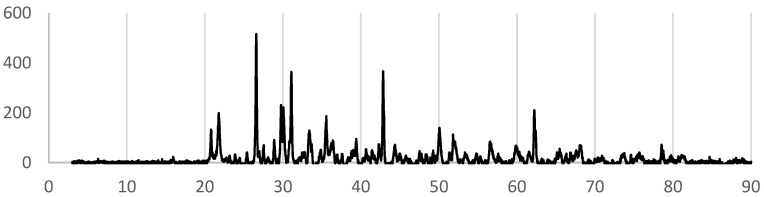
The XRD pattern of the synthesized samples after the phosphorus recovery test.

The rate of decrease in phosphate ions in the 0.25-1300 sample, in which diopside was confirmed, was 70.1%. However, neither the SEM image (Figure 4) nor the XRD pattern (Figure 5) of the 0.25-1300 sample showed any findings on crystal phase that are attributable to phosphorus adsorption.

## 4. Discussion

### 4.1. Synthesis

Diopside synthesis was carried out by introducing Ca and Mg ions, which are essential for diopside formation, into rice husks as a silica source, followed by calcination. The concentration of the Ca–Mg aqueous solution and the calcination temperature were considered the variable parameters to determine the synthesis conditions for diopside. The synthesis was successful at 0.25-1300, that is, with the relatively small Ca and Mg ions in this experimental system and high-temperature calcination.

During the synthesis, when amorphous, highly reactive rice husk silica is immersed in the Ca–Mg aqueous solution, the siloxane bonds in silica undergo cleavage, causing partial dissolution into monosilicate, as expressed by the following reaction [20]:

SiO_2_ + 2H_2_O → Si(OH)_4_

The solubility of rice husk silica is a property relied upon for diopside synthesis. It is known that the dissolution rate of silica increases in the presence of electrolytes [21,22,23,24]. On the other hand, at high electrolyte concentrations, the amount of silica dissolved is supposed to decrease [25,26,27,28,29,30]. As discussed above, if the concentration of electrolytes affects silica dissolution and precipitation, it should alter the silica content and mineral composition within the rice husks. In this case, it can be inferred that the dissolution of silica and subsequent precipitation were more pronounced in rice husks treated with the 2.0 mol/L Ca–Mg aqueous solution. Notably, the successful synthesis of diopside was achieved under the Ca–Mg aqueous solution with lower concentration. Based on the above reasoning, the Ca-Mg aqueous solution with a lower concentration would suppress both the dissolution and precipitation of rice husk silica. In other words, the rice husks treated with the 0.25 Ca-Mg aqueous solution would contain more native silica than that treated with the 2.0 solution. Then, the successful diopside synthesis in this method could be partly attributable to high content of native silica in the rice husks after chemical treatment. Although this result might simply be due to differences in silica content within the rice husks, the diopside synthesis system using rice husks would be improved by proper understanding and control of the material composition right before calcination.

### 4.2. Crystalline Silica Produced by Calcination

Calcination of rice husks often results in the formation of highly crystalline silica, which is a potential health risk. For example, asbestos-like crystalline silica has been associated with an increased risk of lung cancer. However, calcination is necessary not only for diopside synthesis but also for volume reduction in rice husks, utilization of combustion heat, and recycling of rice husk ash. Thus, safety measures for handling crystalline silica must be considered. Several rice husk calcination methods have been developed to suppress crystalline silica formation, though these often require specialized equipment.

In this study, cristobalite and quartz were formed during diopside synthesis. The formation of them is one of negative aspects in rice husk calcination and controlling of the formation is an active issue. On the other hand, cristobalite and quartz formation was not detected for 0.25-1000, which exhibited a possible broad peak for diopside. Therefore, in this synthesis system, it could be possible to find calcination conditions that does not form cristobalite and quartz between 1000 and 1300 °C.

Under synthesis conditions where diopside formation was not observed, other than 0.25-1300 and 0.25-1000, cristobalite was also not detected. Notably, under the conditions for 2.0-600, the overall diffraction intensity was low. Even if the peak near 43° corresponds to forsterite, its impact appears to be minor. While the conditions for 2.0-600 were not favorable for diopside synthesis, they may still be safe and convenient for incinerating rice husks, given the absence of crystalline silica formation.

Under synthesis conditions where diopside formation was not observed, cristobalite and quartz were also not detected. While the conditions other than 0.25-1300 were not favorable for diopside synthesis, they may still be safe and convenient for incinerating rice husks, given the absence of crystalline silica formation.

### 4.3. Phosphorus Recovery Ability of 0.25-1300

In this study, diopside was successfully synthesized under the conditions for the 0.25-1300 sample. Further, phosphorus recovery using the obtained diopside compound was confirmed. However, further examination is required regarding the practicality of its phosphorus recovery ability.

First, the diopside content in the synthesized product must be considered. Based on XRD peak intensity, the diopside content in the final product appears to be low. The final product also contains other minerals and non-crystalline substances in significant quantities. Accordingly, it cannot be denied that components other than diopside also contribute to phosphorus recovery. In other words, the necessity of diopside for phosphorus recovery should be examined. In cases where the contribution of diopside to phosphorus recovery is low, the energy required to repurpose rice husks as phosphorus recovery material can be reduced.

Next, the persistence of diopside in phosphorus recovery must be addressed. In artificial bone applications, diopside interacts with surrounding phosphorus to precipitate calcium phosphate, such as apatite. However, this precipitation is likely limited to the diopside surface, suggesting that once the surface is covered with apatite, diopside itself no longer participates directly in further reactions. As a result, its phosphorus recovery ability may saturate quickly. In artificial bone applications, diopside’s primary role is to promote early calcification, and its short-lived functionality is still considered beneficial. However, in case of phosphorus recovery in hydrosphere environments, its limited functional duration may not justify the cost of implementation.

As a result, while we confirmed the synthesis of diopside using rice husks and phosphorus recovery using the synthesized material, we also revealed the potential challenges of this strategy. However, given that this study focuses on utilizing waste materials such as rice husks, it might still be valuable to harness advantages such as low cost and reduced environmental impact, even if the expected functionality is limited. Future research should focus on increasing the diopside content in the synthesized product while also exploring operational strategies to integrate rice husk-derived diopside into sustainable resource cycles.

## 5. Conclusions

In this study, diopside was successfully synthesized by immersing *Aichi no Kaori* rice husks in a Ca–Mg aqueous solution followed by calcination. The diopside synthesis was successful in a relatively low-concentration Ca–Mg aqueous solution. Cristobalite and quartz formation was also confirmed under the most successful diopside synthesis conditions in this study. However, it was suggested that the formation of them could be suppressed by reducing the calcination temperature and increasing the Ca–Mg aqueous solution concentration. The phosphorus recovery ability of the synthesized diopside compounds was confirmed. Given the diopside content of the compound, it was considered possible that components other than diopside might also be contributing to the phosphorous recovery.

## Data Availability

The original contributions presented in this study are included in the article. Further inquiries can be directed to the corresponding author.

## Abbreviations

The following abbreviations are used in this manuscript:

XRD	X-ray diffraction
SEM	Scanning electron microscopy
